# 

**Published:** 1890-10

**Authors:** 


### CONTENTS

ORIGINAL COMMUNICATIONS—

Tracheotomy for Foreign Bodies in the Air Passages. W. F. Westmoreland, M. D. .......... 449

General Remarks on Some of the Pathological Conditions of the Naso-Pharynx. A. B. Patterson, M. D. .......... 452

Report of Five Laparotomies. X. O. Werder, M. D. .......... 456

Preliminary Report Concerning the Post Mortem Changes in the First Person Executed by Electricity. E. C. Spitzka, M. D. .......... 460

SOCIETY REPORTS—

Richmond Academy of Medicine and Surgery .......... 462

Allegheny County Medical Society .......... 470

Scientific Proceedings of the Richmond Academy of Medicine and Surgery .......... 474

Atlanta Society of Medicine .......... 481

CORRESPONDENCE—

Our New York Letter .......... 485

BOOK REVIEWS—

Intestinal Diseases of Children. Volume I. By A. Jacobi, M. D. .......... 514

EDITORIAL—

The Treatment of Acute Intestinal Obstruction .......... 490

The Atlanta Society of Medicine .......... 493

Ingrowing Toe-Nail .......... 494

Southern Doctors and their Work .......... 495

Personals .......... 496

Electrical Execution .......... 497

Items .......... 493, 498-512

Index to Advertisements on Page xix.

Reading Notices on Page xviii.

Premium List on Pages xxxvi and xxxvii.

